# Monitoring Treatment Efficacy of Antiangiogenic Therapy Combined With Hypoxia-Activated Prodrugs Online Using Functional MRI

**DOI:** 10.3389/fonc.2021.672047

**Published:** 2021-04-30

**Authors:** Mengjie Ma, Jianye Liang, Dong Zhang, Xi Xu, Qingqing Cheng, Zeyu Xiao, Changzheng Shi, Liangping Luo

**Affiliations:** ^1^ Medical Imaging Center, The First Affiliated Hospital of Jinan University, Guangzhou, China; ^2^ Department of Medical Imaging, Sun Yat-sen University Cancer Center, State Key Laboratory of Oncology in South China, Collaborative Innovation Center for Cancer Medicine, Guangzhou, China

**Keywords:** IVIM-DWI, BOLD-MRI, antiangiogenic therapy, hypoxia-activated prodrugs, hypoxia

## Abstract

**Objective:**

This study aimed to investigate the effectiveness of intravoxel incoherent motion (IVIM) diffusion-weighted imaging (DWI) and blood oxygen level-dependent (BOLD) magnetic resonance imaging (MRI) in monitoring tumor responses to antiangiogenic therapy combined with hypoxia-activated prodrugs (HAPs).

**Materials and methods:**

Establishing colon cancer xenograft model by subcutaneously injecting the HCT116 cell line into BALB/C nude mice. Twenty-four tumor-bearing mice were randomly divided into four groups and injected with bevacizumab combined with TH-302 (A), bevacizumab (B), TH-302 (C), or saline (D) on days 1, 4, 7, 10 and 13. Functional MRI was performed before and at 3, 6, 9, 12 and 15 days after treatment. Pathologic examinations, including HE staining, HIF-1α and CD31 immunohistochemical staining, and TUNEL and Ki-67 immunofluorescent staining, were performed after the last scan.

**Results:**

At the end of the study, Group A showed the lowest tumor volume, followed by Groups B, C, and D (F=120.652, P<0.001). For pathologic examinations, Group A showed the lowest percentage of CD31 staining (F=73.211, P<0.001) and Ki-67 staining (F=231.170, P<0.001), as well as the highest percentage of TUNEL staining (F=74.012, P<0.001). Moreover, the D* and f values exhibited positive correlations with CD31 (r=0.868, P<0.001, and r=0.698, P=0.012, respectively). R2* values was positively correlated with HIF-1α (r=0.776, P=0.003). D values were positively correlated with TUNEL (r=0.737, P=0.006) and negatively correlated with Ki-67 (r=0.912, P<0.001). The standard ADC values were positive correlated with TUNEL (r=0.672, P=0.017) and negative correlated with Ki-67 (r=0.873, P<0.001).

**Conclusion:**

Anti-angiogenic agents combined with HAP can inhibit tumor growth effectively. In addition, IVIM-DWI and BOLD-MRI can be used to monitor the tumor microenvironment, including perfusion, hypoxia, cell apoptosis and proliferation, in a noninvasive manner.

## Introduction

Due to the heterogeneity between or within tumors, the efficacy of conventional cytotoxic agents remains limited. Targeting the tumor vasculature is another strategy for cancer therapy, especially vascular targeting therapy. The vascular supply of oxygen and nutrients is indispensable for tumor growth. Without vasculature, tumors cannot grow, invade or metastasize. Vascular targeting therapy has been proven to suppress tumor growth (1, 2). Bevacizumab is an antiangiogenic agent that can bind to VEGF-A specifically and reduce the tumor neovascularization. In a recent study, Shi et al. (2) found that a high dose of VEGF antibody reduces tumor vessels and produces a strong hypoxic response. A hypoxic microenvironment can induce tumor cell apoptosis and necrosis, but tumor cells do not passively suffer from oxygen deprivation. In a hypoxic microenvironment, the expression of angiogenic factors (e.g., VEGF) would be upregulated in tumor cells in order to promote angiogenesis, which may result in treatment failure. A hypoxic microenvironment may increase metastasis risks by inducing epithelial-mesenchymal transition. hypoxia-activated prodrug (HAPs) are a category of nontoxic or hypotoxic drugs under normoxic conditions, but these drugs can be activated in a hypoxic microenvironment. TH-302 (evofosfamide) is the second-generation HAP that can be activated under hypoxic conditions by the reduction of its 2-nitroimidazole moiety and release the toxic effector bromo-isophosphoramide mustard (Br-IPM), which can crosslink DNA, leading to tumor cell death (3).

To monitor the therapeutic efficacy of anti-vascular therapy in real time, noninvasive MRI is an essential part of our study. intravoxel incoherent motion (IVIM) diffusion-weighted imaging (DWI) has been used to assess tumor perfusion in recent years and shows good coherence to pathological markers (1). The IVIM biexponential model is the most widely used model and can separate the diffusion motion of intercellular water molecules into the pure diffusion fraction (D) and the diffusion motion of intravascular water molecules into the perfusion-dominated “pseudodiffusion” fraction (D*) (4). IVIM-DWI can evaluate tumor microperfusion without the need for injection of contrast agent, which mean that it can be performed at short time interval. Aside from perfusion changes, the degree of hypoxia is another noteworthy feature during antivascular therapy. Blood oxygen level-dependent (BOLD) magnetic resonance imaging (MRI) was originally developed by Ogawa (5) in 1990, who found that paramagnetic deoxyhemoglobin in blood is an endogenous contrast agent that can provide real-time maps of blood oxygenation in the brain *in vivo*. BOLD-MRI was originally used to reflect the blood oxygen level in the brain. In addition, recent studies suggest that BOLD-MRI can be used to monitor the hypoxia microenvironment of tumor (6).

In this study, our primary objective was to investigate whether IVIM-DWI and BOLD-MRI could be used to describe the changes in tumor microperfusion and hypoxic during antiangiogenic therapy. Furthermore, we wanted to examine whether TH-302 could act synergistically with bevacizumab by targeting the hypoxic regions resulting from antiangiogenic therapy.

## Materials and Methods

### Human Colon Cancer Xenografts

All animal experiments were performed in accordance with procedures approved by the Animal Care and Use Committee of our institution. Female BALB/c nude mice were supplied by Beijing Huafukang Bioscience Co. Inc. (Beijing, China). The human colon cancer HCT116 cell line obtained from the American Type Culture Collection (ATCC, Manassas, Virginia) was used to develop a tumor model. Colon cancer xenograft models were established by subcutaneously injecting 0.2 ml of 1×10^6^/ml cells into the front legs of mice. Tumors reach approximately 200 mm^3^ in tumor volume and cost approximately 2 weeks.

### Treatment and Grouping

A total of 24 tumor-bearing mice were randomly divided into 4 groups (six for each): treated with (A) bevacizumab (10 mg/kg, Roche, Switzerland) and TH-302 (50 mg/kg, MedChemExpress, USA), (B) bevacizumab (10 mg/kg), (C) TH-302 (50 mg/kg), or (D) saline. All mice were treated by intraperitoneal injection on days 1, 4, 7, 10 and 13. Tumor volumes were measured using calipers on days 0, 3, 6, 9, 12 and 15 after treatment and calculated using the formula: (short diameter)^2^×(long diameter)×0.5.

### MRI Acquisition

MR scanning were performed with a 1.5 T Signa HDxt superconductor clinical MR system (GE Medical System, Milwaukee, WI). All mice in each group were scanned equipped with a special animal coil under anesthesia. Conventional T1-weighted images (T1WI) were acquired using fast spin-echo (FSE) sequences [repetition time/echo time (TR/TE) = 340/14.7 ms, field of view (FOV) = 5×5 cm^2^, matrix size = 192×160, slice thickness = 2 mm, slice gap = 0.2 mm, number of excitations (NEX) = 2]. T2-weighted images (T2WI) were acquired using fast-recovery fast spin echo sequences [TR/TE = 1900/82.3 ms, FOV = 5×5 cm^2^, matrix size = 256×192, slice thickness = 2 mm, slice gap = 0.2 mm, NEX = 2]. IVIM-DWI MRI was acquired using a single-shot, echo-planar imaging pulse sequence [TR/TE = 3000/101.7 ms, FOV= 5×5 cm^2^, matrix size = 128×96, slice thickness = 2 mm, slice gap = 0.2 mm, diffusion gradients applied in three orthogonal directions with 13 b values: 0, 25, 50, 75, 100, 150, 200, 400, 600, 800, 1000, 1200, 1500 s/mm^2^, and NEX of 3 for each b value] with chemical shift-selective saturation technique for fat suppression. BOLD-MRI was acquired using a three-dimensional spoiled gradient echo sequence [TR = 160 ms, TE = 3.4, 9.3, 15.2, 21.2, 27.1, 33, 38.9, 44.8, 50.7, 56.6, 62.5, 68.5, 74.4, 80.3, 86.2, 92.1 ms, FOV = 8.0×6.4 cm^2^, matrix size = 192×128, slice thickness = 2 mm, slice gap = 0.2 mm, NEX = 2].

### Image Analysis

All of the MR functional images were processed using a postprocessing workstation (AW4.5, GE Healthcare). We delineated the tumor border as regions of interest (ROIs) on the largest cross section of the tumor. We analyzed the IVIM-DWI date with biexponential model: S_b_/S_0_= (1-f) ×exp^-b×D^ +f×exp^-b×D*^ by Functool-MADC software. The S_b_ in the formula represents the signal intensity of the ROI at different b values, and S_0_ is the average signal intensity for a b value of 0. The D values represent the true diffusion coefficient, D* represents the pseudodiffusion coefficient, and f is the perfusion fraction. The apparent diffusion coefficient (ADC) values were calculated by using all b values with a monoexponential fit. BOLD-MRI data were analyzed using Functool-R2Star software. A single exponential model of the ln(signal intensity) to the TE curve was linearly fitted to generate the R2* maps. The R2* (1/T2*) values were determined by the slope of ln(signal intensity) versus TE (7).

### Histological Analysis

After the final time point of MR scanning (corresponded to day 15), three mice were randomly selected from each group for pathologic examinations. Pathologic examinations included hematoxylin and eosin (HE) staining, HIF-1α and CD31 immunohistochemical staining, terminal deoxynucleotidyl transferase-mediated dUTP nick end labeling (TUNEL) and Ki-67 immunofluorescent staining. Tumors were completely dissected from tumor-bearing mice, fixed with 4% paraformaldehyde, embedded in paraffin, then sliced into 5-μm thick sections and stained with HE according to standard procedure. The antibodies needed in this study were acquired from Servicebio Technology Co., Ltd. (Wuhan, China). HIF-1, CD31, TUNEL and Ki-67 stains were used to identify tumor hypoxia, blood vessels, DNA damage and cell proliferation, respectively. Pathological sections were visualized and recorded by an Olympus BX 53 microscope. Three typical fields (× 200) were selected from each section, and measured the integrated optical density (IOD) of the positive staining region by Image-Pro Plus 6.0 software (Media Cybernetics, USA).

### Statistical Analysis

All the statistical date were analyzed by using SPSS 22.0 software (IBM Corporation, USA), and the statistical graph as shown were made by GraphPad Prism 8.0 (GraphPad Software Inc., USA). The numeric dates shown in our article are presented as the mean ± standard deviation (SD). The Kolmogorov–Smirnov test was used to evaluate the data distribution type. One-way analysis of variance (ANOVA) with least significant difference (LSD) as a *post hoc* test was used to analyze the IVIM-DWI and BOLD-MRI parameters, tumor volume, and pathologic markers in each group at different time points or between groups at each time point. The correlation between pathologic markers and the parameters of functional MRI at the last time point was evaluated using Pearson correlation analysis. Statistically significant was considered when P<0.05.

## Results

### Treatment Efficacy of Tumor Growth

**Figure 1:**
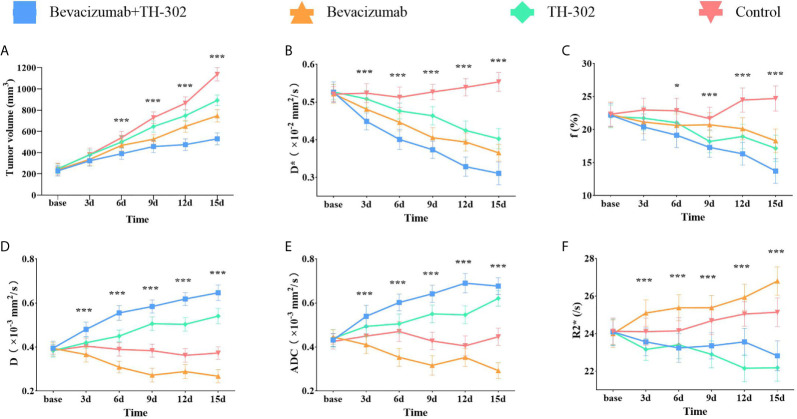
Tumor growth curves **(A)** and longitudinal monitoring of functional MRI parameters D* **(B)**, f **(C)**, D **(D)**, ADC **(E)** and R2* **(F)** values at different time points in the 4 groups. Dates presented as the mean SD. *P < 0.05, **P < 0.01 and ***P < 0.001 represent the analysis results.

Tumor growth manifested a significant difference between groups as early as day 6 (F=8.375, P=0.001) in this study. The treatment groups (including bevacizumab, TH-302 and bevacizumab + TH-302) exhibited smaller tumor volumes than the control group (**Figure 1A**). At day 15, the tumor volume in Group A was the lowest, followed by that in Groups B, C, and D (F=120.652, P<0.001). The tumor growth inhibitory rates were 53.4% (Group A), 34.3% (Group B), and 21.5% (Group C).

### Treatment Efficacy Assessed by MRI

**Figure 2:**
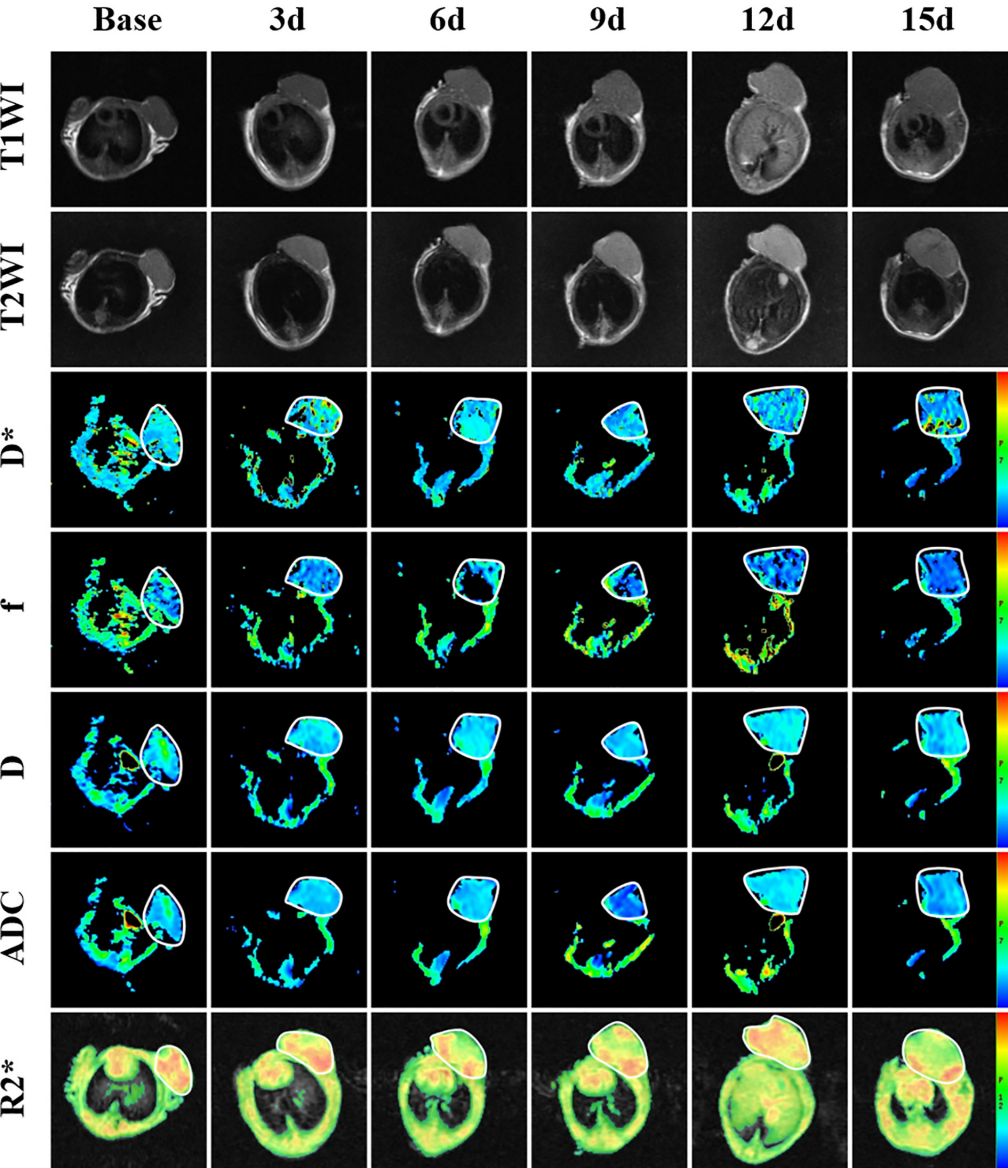
T1-weighted image, T2-weighted image and parametric maps (D*, f, D, ADC and R2*) of a representative mouse treated with bevacizumab combined with TH-302 before and at different times after treatment.

To monitor tumor responses in the tumor microenvironment, including microperfusion and hypoxia levels, we performed IVIM-DWI and BOLD-MRI on the 4 groups longitudinally. The pseudocolor maps (D*, f, D and R2*) of Group A at each time point are shown in **Figure 2**. The longitudinal measurements of functional MRI parameters (D*, f, D and R2*) in Group A at each time point are shown in **Table 1**.

**Table 1:** Longitudinal measurements of functional MRI parameters and tumor volume in Group A.

Group A	base	3 days	6 days	9 days	12 days	15 days	F	P
D* (10^-2^mm^2^/s)	0.526 ± 0.027	0.449 ± 0.022	0.401 ± 0.026	0.374 ± 0.023	0.329 ± 0.025	0.311 ± 0.030	57.248	0.001
f (%)	22.2 ± 1.7	20.4 ± 2.0	19.2 ± 1.9	17.3 ± 1.5	16.3 ± 1.7	13.7 ± 1.9	17.319	0.001
D (10^-3^mm^2^/s)	0.395 ± 0.032	0.481 ± 0.033	0.556 ± 0.033	0.585 ± 0.030	0.619 ± 0.029	0.647 ± 0.035	51.177	0.001
ADC (10^-3^mm^2^/s)	0.432 ± 0.029	0.540 ± 0.050	0.602 ± 0.038	0.642 ± 0.039	0.690 ± 0.044	0.677 ± 0.038	35.341	0.001
R2* (s^-1^)	24.093 ± 0.736	23.563 ± 0.741	23.237 ± 0.765	23.354 ± 0.699	23.561 ± 0.720	22.819 ± 0.798	1.932	0.118
Tumor volume (mm^3^)	228.7 ± 49.2	323.5 ± 50.8	389.8 ± 52.4	457.1 ± 58.0	475.4 ± 54.9	529.8 ± 56.6	25.373	0.001

The F and P values represent the analysis results of one-way ANOVA.

The variation tendencies of perfusion-related parameters (D*, f) of IVIM-DWI are shown in **Figures 1B, C**. The perfusion-related parameters gradually decreased in all 3 treatment groups (bevacizumab, TH-302 and bevacizumab + TH-302). The D* values exhibited a significant difference between groups (F=11.225, P<0.001) as early as day 3, and the f values exhibited a significant difference on day 6 (F=4.413, P=0.015). For Group A, the mean values of D* and f on day 15 decreased to 59.1% and 61.7% of their baseline, respectively. On day 15, Group A exhibited the lowest D* values, followed by Groups B, C, and D (F=93.838, P<0.001), and the lowest f values, followed by Groups C, B, and D (F=35.741, P<0.001).

As shown in **Figures 1D, E** the standard ADC values and diffusion-related parameter D gradually increased in Groups A and C and decreased in Group B. Significant difference initially appeared on day 3 in both ADC and D values (F=8.685, P<0.001 and F=12.649, P<0.001, respectively). For Group A, the standard ADC values on day 15 increased to 156.7% of its baseline, and the D values increased to 163.8%. On day 15, Group A exhibited the highest ADC and D values (F=131.492, P<0.001 and F=166.729, P<0.001, respectively).To monitor hypoxia levels, we performed BOLD-MRI as a noninvasive method. As shown in **Figure 1F**, the R2* values exhibited a slight decrease in Group A, but no significant difference was observed (F=1.932, P=0.118). R2* values exhibited an uptrend in Groups B and D and a downtrend in Group C. On day 15, Group B exhibited the highest R2* values, followed by Groups D, A, and C (F=47.299, P<0.001).

### Treatment Efficacy Assessed by Histology

**Figure 3:**
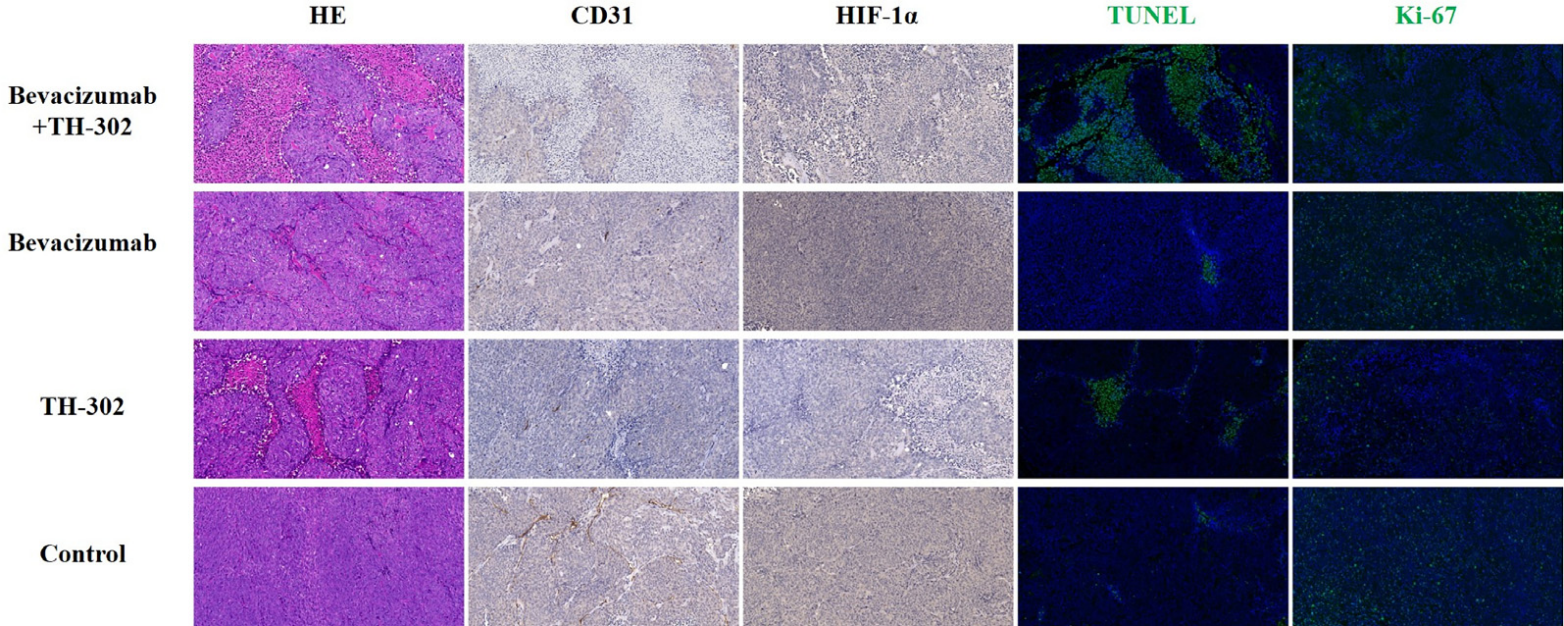
Representative HE (× 200), CD31 (× 200), HIF-1α (× 200), TUNEL (× 200) and KI-67 (× 200) staining of the 4 groups at the end of the experiment.

At the end of the experiment, we performed CD31, HIF-1α, TUNEL, and Ki-67 assays to assess MVD, hypoxic degree, tumor cell apoptosis, and tumor cell proliferation, respectively. Representative pathological staining sections of the 4 groups as shown in **Figure 3**. For CD31 staining, Group A showed the lowest percentage of CD31 staining, followed by Groups B, C, and D (F=73.211, P<0.001). For HIF-1α staining, Group C showed the lowest percentage of HIF-1α staining, followed by Groups A, D and B (F=46.465, P<0.001). For TUNEL staining, Group A showed the highest percentage of TUNEL staining, followed by Groups C, B, and D (F=74.012, P<0.001). For Ki-67 staining, Group A showed the lowest percentage of Ki-67 staining, followed by Groups C, D, and B (F=231.170, P<0.001). Tumors treated with bevacizumab and TH-302 had less MVD, a lower degree of hypoxia, more necrosis, and less proliferation than control tumors (all P values were less than 0.05).

### Correlations Between Functional MRI Parameters and Pathologic Markers

**Figure 4:**
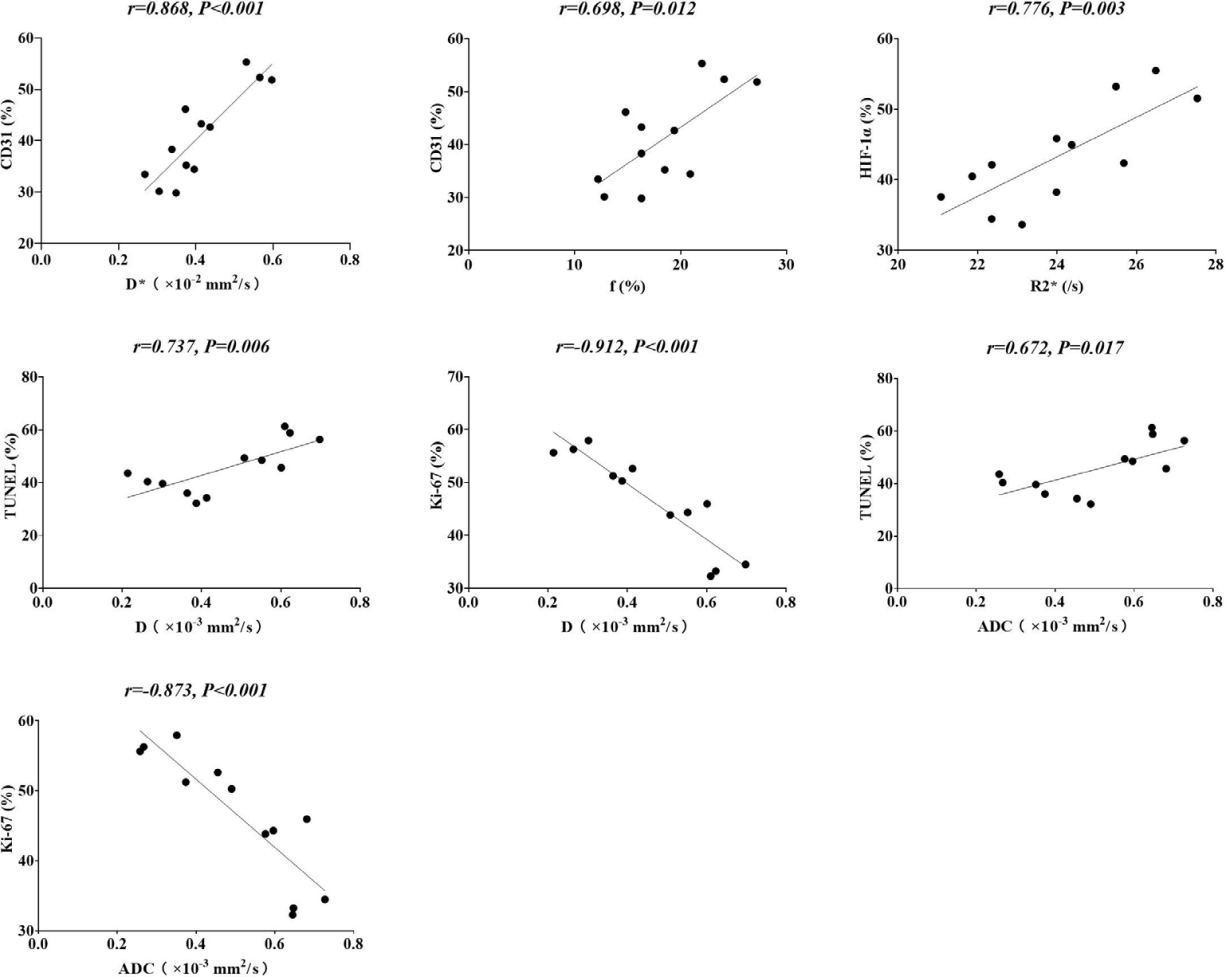
Correlations between functional MRI parameters and pathologic markers.

To verify the efficiency of IVIM-DWI and BOLD-MRI in reflecting the tumor microenvironment, including microperfusion and hypoxia degree, we tried to determine the correlation between MRI and pathological indicators. As shown in **Figure 4**, for tumor microperfusion, the perfusion-related parameters D* and f values exhibited positive correlations with CD31 (r=0.868, P<0.001, and r=0.698, P=0.012, respectively). For the degree of tumor hypoxia, the R2* values showed a positive correlation with HIF-1α (r=0.776, P=0.003). For tumor cell necrosis and proliferation, diffusion-related parameter D revealed a positive correlation with TUNEL (r=0.737, P=0.006) and a negative correlation with Ki-67 (r=0.912, P<0.001). Besides that, the standard ADC values was positive correlated with TUNEL (r=0.672, P=0.017) and negative correlated with Ki-67 (r=0.873, P<0.001) as well.

## Discussion

Tumor vessels play an indispensable role in the growth, invasion and metastasis of solid tumors (8). Tumor cells obtain oxygen and nutrients from tumor vessels, which is also an important metastatic passage (9). Anti-angiogenesis in tumor therapy is a well-known idea that was proposed by Folkman in 1971 (10). Nevertheless, the anti-angiogenesis treatment efficacy was not as good as expected (11). The hypoxic microenvironment caused by VEGF(R) inhibitors can stimulate metastasis (12), which may result in treatment failure. In this study, we aimed to target an anti-VEGF agent (bevacizumab)-induced hypoxic area by applying HAP (TH-302). The first goal of this study was to explore the treatment efficacy of bevacizumab and TH-302 compared with monotherapy. In our study, tumors treated with bevacizumab and TH-302 exhibited lower tumor volumes than tumors in the monotherapy and control groups as well as the largest apoptosis area confirmed by TUNEL staining, indicating that bevacizumab combined with TH-302 can inhibit tumor growth effectively by increasing cell apoptosis. In our study, bevacizumab monotherapy exhibited the largest area of hypoxia and cell proliferation, which was confirmed by HIF-1α staining and Ki-67 staining. A previous study found that HIF-1α expression was positively correlated with Ki-67 (13), and our results were consistent with this finding. When tumor cells are exposed to hypoxic conditions, signaling pathways that regulate proliferation are activated (14). Hypoxia propels tumor progression by facilitating genomic instability of tumor cells, and inactivation of the cell apoptosis pathway is indispensable for tumor cell survival during this procedure (15). Inactivation of the BAX/BAK apoptotic pathway is essential for tumor growth, and cells that express functional BAK and BAX could not survive under hypoxic conditions (16). Ki-67 affects cell cycle progression and is well connected with cell proliferation. The high level of Ki-67 expression predicted a poor clinical prognosis and worse survival (17). In the long run, monotherapy with bevacizumab cannot inhibit tumorigenesis. In our study, the combination group showed the lowest cell proliferation compared with the other 3 groups, as confirmed by Ki-67 staining, indicating that antiangiogenic agents combined with HAP can inhibit tumor growth more effectively without stimulating the proliferation of tumor cells.

Changes in tumor physiology, including perfusion and hypoxia, always occur earlier than changes in morphology. Studies about evaluating *in vivo* tumor perfusion had been widely reported. Photoacoustic imaging is an emerging quantitative manner in many medical imaging modalities, which can detect angiogenesis by estimating wall shear rate (18). High resolution, sufficient imaging depth make photoacoustic imaging have great potential for clinical translation. However, reproducibility and standardization are the challenges for its popularization (19). MRI is a widely used modality for imaging tumor perfusion. Hence, we aimed to monitor the tumor response to antiangiogenic agents combined with HAP using functional MRI. A previous study suggested that the parameters of IVIM-DWI were well correlated with dynamic contrast-enhanced MRI (DCE-MRI) (1). IVIM-DWI is a potential substitute for DCE-MRI in assessing tumor microperfusion without needing of contrast agent. Thus, we applied IVIM-DWI to monitor tumor microperfusion longitudinally. Our research findings were consistent with a previous study, and both the D* and f values exhibited positive correlations with CD31, demonstrating that the D* and f values of IVIM-DWI can be used to assess tumor microperfusion. The D* value, which is also known as the pseudodiffusion coefficient, is closely related to microvascular blood velocity; the f value, which is also called the perfusion fraction, goes hand in hand with the microvascular blood volume fraction (20). D* is considered a surrogate marker of capillary perfusion (21), and the f value is related to the perfusion fraction of the whole diffusion motion (22). Both D* and f were connected with perfusion characteristics, and the results of our study also supported this. Furthermore, the D values showed a positive correlation with TUNEL and a negative correlation with Ki-67, which was consistent with previous reports (23, 24). These results indicate that the D values can be applied to evaluate tumor cell apoptosis and proliferation in a noninvasive way. The D values were related to diffusion motion of intercellular water molecules, which would be limited by the irregular and tight arrangement of tumor cells. These highly proliferative tumor cells turn into necrotic areas after treatment with bevacizumab combined with TH-302, and the limitation of water molecules is reduced. Interestingly, the standard ADC in our study was well correlated with TUNEL and Ki-67, which were similar to the D values of IVIM, indicating that it could be a suitable marker for the assessment of tumor cell apoptosis and proliferation. However, there was no statistically significant correlation between standard ADC values and CD31 (P=0.516), which leaded to its limitations in our study. Moreover, the R2* values of BOLD-MRI showed a positive correlation with HIF-1α, indicating that the R2* values are a promising parameter to predict tumor hypoxia. In this study, significant differences in the D, D* and R2* values between the 4 groups were found as early as day 3; in contrast, significant differences in tumor volume were found at day 6. Our study suggests that functional MRI can predict the therapeutic effect earlier than morphology.

Our study has some limitations. First, we injected bevacizumab and TH-302 simultaneously in this study. TH-302 is a drug that can selectively damage hypoxic cells, and the efficacy of this drug is increased when tumors are exposed to high levels of hypoxia. On the other hand, bevacizumab can inhibit angiogenesis of tumors, which may hamper delivery of TH-302 to the tumor. However, this is beyond the scale of our research. Second, we did not perform DCE-MRI to assess tumor perfusion after antivascular therapy. However, a previous study demonstrated that IVIM-DWI could represent an alternative method to DCE-MRI. It is still uncertain that which pattern is more suitable for monitoring tumor perfusion. Thirdly, the standard ADC has the potential to evaluate tumor cells necrosis and proliferation, but could not evaluate tumor perfusion simultaneously.

In conclusion, our results suggest that antiangiogenic agents combined with HAP produce a strong inhibitory effect on tumor growth. In addition, IVIM-DWI and BOLD-MRI can be used to monitor the tumor microenvironment, including perfusion, hypoxia, cell apoptosis and proliferation, in a noninvasive manner. D, D* and R2* values can predict the therapeutic effect of antiangiogenic agents combined with HAP earlier than morphology.

## Data Availability Statement

The original contributions presented in the study are included in the article/supplementary material. Further inquiries can be directed to the corresponding authors.

## Ethics Statement

The animal study was reviewed and approved by Institute of Laboratory Animal Science, Jinan University.

## Author Contributions

The study concepts were designed by ZX, CS, and LL. Date acquired and analyzed by MM and DZ. JL, XX, and QC contributed to quality control of data and statistical analysis. Manuscript was prepared and wrote by MM. Manuscript reviewed and edited by JL. All authors contributed to the article and approved the submitted version.

## Funding

This work was supported by the National Natural Science Foundation of China [Grant Number 81771973, 81971672]; the Guangzhou Key Laboratory of Molecular and Functional Imaging for Clinical Translation [Grant Number 201905010003]; the Engineering Research Center of Medical Imaging Artificial Intelligence for Precision Diagnosis and Treatment, Guangdong Province; the Key Program of the Natural Science Foundation of Guangdong Province [Grant Number 2018B0303110011]; the Fundamental Research Funds for the Central Universities [Grant Number 21620308, 21620101].

## Conflict of Interest

The authors declare that the research was conducted in the absence of any commercial or financial relationships that could be construed as a potential conflict of interest.
